# Managing Subarachnoid Hemorrhage Precipitated by Anesthesia-assisted Rapid Opioid Detoxification: A Case Report

**DOI:** 10.5811/cpcem.2021.9.53868

**Published:** 2022-01-06

**Authors:** Michael F. Sobin, Liem Ngo, Danielle Turner Lawrence, Abigail Kirwen

**Affiliations:** Beaumont Health-Royal Oak, Department of Emergency Medicine, Royal Oak, Michigan

**Keywords:** case report, anesthesia, opioid withdrawal, subarachnoid hemorrhage

## Abstract

**Introduction:**

Anesthesia-assisted rapid opioid detoxification (AAROD) is a controversial method of treating opioid dependence that involves sedating patients during a period of provoked withdrawal. Reported adverse outcomes of the procedure demonstrate the importance of recognizing the potential complications of AAROD.

**Case Presentation:**

We present a case of a 41-year-old male presenting with a subarachnoid hemorrhage following an AAROD procedure.

**Conclusion:**

This case report and discussion reviews the pathophysiology of opioid withdrawal syndrome, potential complications following AAROD, and important management considerations.

## INTRODUCTION

Emergency department (ED) visits and hospitalizations for opioid withdrawal have occurred at an increasing rate over the past 20 years.^1^ Treatment of symptomatic withdrawal and opioid dependence have been subjects of research for the past three decades.^2^ One such method involves acutely precipitating withdrawal with opioid antagonists while ameliorating the symptoms using sedatives, a treatment known as anesthesia-assisted rapid opioid detoxification (AAROD). This treatment method, while sought after for its perceived ease and short course, has been found to provide inconsistent rates of opioid abstinence while being costlier and riskier compared to available alternatives.^2^ Additionally, little is known about how to manage adverse effects of AAROD in the ED setting. Here, we present a case describing the emergent treatment of a patient with subarachnoid hemorrhage after AAROD.

## CASE REPORT

A 41-year-old male with history of opioid and cocaine use disorders was brought to the ED after being found unresponsive at an opioid detoxification center. The day prior to admission, the patient underwent an observed AAROD where he received buprenorphine while undergoing general anesthesia. After completion of treatment the patient was transferred to a local center where he was observed by onsite staff, receiving as-needed benzodiazepines for any continued withdrawal symptoms. Subsequently, the patient began to complain of a severe headache and ataxia and exhibited agitated behavior. Early the following morning he was found unresponsive and in respiratory distress. Emergency medical services administered naloxone without a change in his condition. A laryngeal mask airway was placed, and he was transported to the ED.

Upon arrival, the patient’s vitals were heart rate 152 beats per minute, blood pressure 162/101 millimeters (mm) of mercury, respiratory rate 64 breaths per minutes, oxygen saturation 100% on 15 liters of oxygen via a nonrebreather, and temperature 37.9 Celsius. The patient was diaphoretic and had a Glasgow Coma Scale score of three. His head was atraumatic, and his pupils were 3 mm and reactive bilaterally. He had significant upper airway secretions, and lung auscultation revealed diffuse bilateral rhonchi. His peripheral pulses were bounding and his skin was mottled and pale. There was no evidence of traumatic injury. After he failed to improve with an additional dose of naloxone, the patient was sedated and intubated.

An initial electrocardiogram (ECG) revealed supraventricular tachycardia that failed to convert with administration of multiple doses of adenosine. Initial labs were notable for a pH of 7.17, lactic acid of 8.0 millimoles per liter (mmol/L) (reference range: 0.5 – 2.0 mmol/L), white blood cell count of 37.1 billion per liter (bil/L) (3.5 – 10.1 bil/L) with left shift, and troponin of 16.38 nanograms per milliliter (ng/mL) (<0.3 ng/mL). Chest radiograph was significant for diffuse pulmonary edema. A non-contrast head computed tomography (CT) revealed extensive subarachnoid hemorrhage and right temporal intraparenchymal hemorrhage with 5 mm of midline shift. A CT angiogram revealed a 3-mm right internal carotid artery aneurysm as the source of the bleeding. Treatment with continuous nicardipine infusion was initiated, and neurosurgery placed an external ventricular drain in the ED.

The patient was admitted to the surgical intensive care unit for aneurysmal repair. Further conversation with family indicated no family history suggestive of cerebral aneurysmal disease or known preceding symptoms. His procedure, completed successfully, was complicated by a four-minute episode of pulseless electrical activity cardiac arrest. He subsequently developed a right middle cerebellar artery infarct with evidence of herniation. With poor neurologic prognosis, the family chose comfort measures, and the patient died on hospital day three.

## DISCUSSION

Opioid use disorder in the United States remains at an all-time high, with hospitalization rates increasing 219% between 1998 (58.9 per 100,000) and 2016 (190.7 per 100,000).^1^ Opioid withdrawal is a complicated neurobiological interaction caused by the decreased responsiveness of opioid receptors in the brain from chronic use. In the locus coeruleus, periaqueductal gray and rostral ventromedial medulla the lack of mu opioid response leads to an overwhelming surge of noradrenaline and other monoamines.^3^ These surges produce electrolyte derangements and respiratory alkalosis, which have been linked to episodes of cardiac arrest and seizure in previous opioid withdrawal cases.^4^,^5^ Outside of opioid withdrawal, massive catecholamine surges have been documented as possible precipitants of subarachnoid hemorrhage.^6^ Historically, treatment regimens focused on treating the acute sympathetic overdrive with alpha-2 agonists and benzodiazepines and weaning patients with long-acting opioid agonists such as methadone and buprenorphine.^7^ However, concerns of limited availability and length of treatment have continued to push researchers and clinicians to search for additional strategies.^8^

**CPC-EM Capsule**

What do we already know about this clinical entity?

*Anesthesia-assisted rapid opioid detoxification (AAROD) is a rare treatment method of opioid use disorder with reported significant procedural complications*.

What makes this presentation of disease reportable?

*This is the first reported case in the literature of AAROD complicated by subarachnoid hemorrhage*.

What is the major learning point?

*Treatment of AAROD complications is based on reducing opioid withdrawal symptoms from the pathologic sympathetic surge while providing long-acting opioid agonists*.

How might this improve emergency medicine practice?

*Early recognition and prompt treatment of AAROD complications may lead to improved outcomes in these patients*.

In the 1990s, the combination of difficulties prescribing long-acting opioids and the desire for a faster resolution of opioid withdrawal symptoms led clinicians to investigate opioid withdrawal induction in conjunction with sedation.^8^ The method described, known as AAROD, involves precipitating withdrawal using various opioid antagonists while sedating or anesthetizing patients to mask withdrawal symptoms. Although pre-procedure evaluations are not standardized, a general medical exam, blood work, ECG, and urine drug screen are often employed.^2^ Patients are typically excluded if there has been recent cocaine use or significant medical or psychiatric disease history.^2^ While initial studies did show the effectiveness of AAROD in transitioning patients to opioid abstinence.^9^ over the 20 years of its use additional concerns have been raised on the safety and efficacy of AAROD.^10^–^12^

A randomized clinical trial examining AAROD vs buprenorphine and clonidine alone found equivalent rates of opioid abstinence, while AAROD conferred significantly increased patient risks such as acute psychiatric disturbances, cardiac arrhythmias, and pulmonary edema.^2^ A 2013 report from the US Centers for Disease Control and Prevention detailed one New York City AAROD clinic where seven of 75 patients experienced serious adverse events requiring hospitalization with two resultant deaths.^10^ Additionally, two Cochrane reviews found AAROD to be equally effective compared to traditional treatment options but with significantly higher mortality, out-of-pocket patient cost, and medicolegal risk; the reviews recommended avoidance of the technique.^11^,^12^ Currently, only methadone and buprenorphine have shown consistent evidence for effective treatment of opioid use disorders, with superior rates of long-term opioid abstinence compared to AAROD, abstinence alone, naltrexone, alpha agonists, and benzodiazepines.^2^,^14^ Yet numerous AAROD programs still exist across the country.

Clinicians should maintain a broad differential when approaching critically ill patients after AAROD. Evaluations should focus on potential neurologic, cardiac, electrolyte, and metabolic abnormalities. Reported complications from the procedure include the following: those due to opioid withdrawal symptoms, such as vomiting, diarrhea, hypovolemia and electrolyte abnormalities; those related to adverse effects of the resultant catecholamine surge; and complications arising from general anesthesia.^4^,^5^ Documented adverse outcomes of AAROD include severe psychiatric disturbances, aspiration, cardiac dysthymias, cardiac arrest, respiratory arrest and death.^10^–^12^

In our literature review, we did not find any previously documented cases of subarachnoid hemorrhage following AAROD. Management should focus on reducing withdrawal symptoms, decreasing the catecholamine surge, and providing additional supportive care as necessary. Clonidine has been used to reduce sympathomimetic hyperactivity. Adding short-acting opioid analgesics or increasing the frequency of long-acting opioids are additional options.^13^ Pain management, which can be complicated by the type and level of opioid antagonist used in the AAROD procedure, can be achieved by non-opioid means including intravenous acetaminophen, dexmedetomidine, and gabapentin.^15^ Early utilization of these management options may confer improved outcomes for critically ill post-AAROD patients.

## CONCLUSION

Anesthesia-assisted rapid opioid detoxification is an uncommon and controversial procedure used to treat opioid use disorder. This case of subarachnoid hemorrhage precipitated by AAROD to our knowledge represents the first such documented instance in the literature. Clinicians seeing patients brought to the ED after AAROD should be aware of the potential for serious complications as outlined here. Strategies for managing AAROD complications focuses on supportive care while decreasing withdrawal symptoms and sympathetic surge. In addition, clinicians should remain educated on best practices of opioid use disorder management including methods for using and referring for buprenorphine and methadone treatments.
